# Constructing (101)-Oriented Anatase TiO_2_ Seed Layers on Amorphous Microchannel Plate Glass: Surface Energetics and Template-Assisted Oriented Growth

**DOI:** 10.3390/nano16040281

**Published:** 2026-02-23

**Authors:** Xiang Li, Hua Cai, Wei Wang, Xuan Zhao, Xin-Yue Guo, Meng-Nan Ma, Yue-Yang Zhu, Kai-Ming Li, Hui Liu

**Affiliations:** 1 School of Physical Science and Technology, Guangxi University, Nanning 530004, China; lx2019212911@163.com (X.L.);; 2 China Building Materials Academy, Beijing 100024, China; 3 Key Laboratory of China Building Materials Industry for Special Photoelectric Materials, Beijing 100024, China

**Keywords:** atomic layer deposition (ALD), microchannel plate (MCP), TiO_2_ seed layer, template-assisted oriented growth, surface energy minimization, crystal facet orientation, BaTiO_3_

## Abstract

Integrating functional perovskites on an amorphous microchannel plate (MCP) glass faces challenges regarding the lack of ordered nucleation sites and stringent thermal budgets. Herein, we propose a surface energetics-based atomic layer deposition (ALD) strategy to achieve template-assisted oriented BaTiO_3_ growth via a (101)-oriented anatase TiO_2_ seed layer. Systematic investigation of the TiCl_4_/O_3_ process reveals a kinetic-to-thermodynamic transition at 300 °C, triggering a singular (101) preferred orientation. Combined DFT calculations and Wulff construction elucidate that this texture evolution is governed by a thermally activated surface energy minimization mechanism, driven by the intrinsic stability of the (101) facet. Crucially, the optimized seed layer acts as a multifunctional template: it not only transforms BaTiO_3_ growth from random polycrystalline morphology to a singular (100) orientation with suppressed bulk carbonate impurities but also ensures excellent conformality and uniformity throughout the high aspect ratio microchannels. This study clarifies the thermodynamic mechanism of oriented growth on amorphous substrates, providing a versatile surface engineering pathway for constructing high-performance MCP-based heterojunction devices.

## 1. Introduction

In the rigorous environments of cryogenic physics and deep-space exploration, Microchannel Plates (MCPs) are facing unprecedented performance demands. Under ultra-low temperature conditions, traditional lead silicate glass substrates suffer from the semiconductor ‘freeze-out’ effect, where the bulk resistance increases exponentially, leading to the depletion of strip replenishment current and gain instability [1,2,3,4,5]. To overcome this intrinsic limitation, integrating high-quality functional oxide thin films (such as perovskite-type BaTiO_3_) inside the microchannel arrays is essential to engineer the electrical transport behavior [6,7,8,9,10,11]. However, a formidable obstacle exists: the MCP substrate is typically composed of amorphous lead silicate glass, which lacks long-range ordered lattice templates. Direct growth of complex perovskite materials on such amorphous substrates inevitably leads to random polycrystalline orientation and high-density grain boundary defects [12,13]. These structural imperfections severely scatter charge carriers, further impairing the electrical performance of the device. Therefore, constructing a high-quality intermediate ‘crystalline seed layer’ to induce the template-assisted oriented growth of subsequent functional materials has become key to solving this issue [14,15,16,17].

Although lattice-matched materials like SrTiO_3_ are often used as ideal seed layers for BaTiO_3_ growth [18,19], this traditional strategy faces insurmountable physical bottlenecks in MCP fabrication. First is the stringent Thermal Budget Constraint: the softening point of the MCP glass matrix is typically below 600 °C [20,21], which precludes the high-temperature annealing crystallization processes required for traditional perovskite seed layers; otherwise, it would lead to the structural collapse of the microchannels. Second is the geometric limitation: the extremely High Aspect Ratio (40:1) of MCPs demands deposition techniques with exceptional conformality, rendering line-of-sight deposition techniques such as Pulsed Laser Deposition (PLD) or Sputtering ineffective. Consequently, All-Atomic Layer Deposition (All-ALD) processes have become the only viable pathway [22,23,24,25,26,27,28]. Thus, there is an urgent need to develop an alternative seed layer material that can crystallize at low temperatures (<400 °C) and is compatible with ALD processes to overcome these dual limitations.

Anatase titanium dioxide (TiO_2_), with its relatively low crystallization temperature and mature TiCl_4_/O_3_ ALD chemistry, stands out as an ideal candidate to resolve this dilemma [26,29,30,31]. In particular, the (101) facet, known for its high surface activity [32,33], can theoretically serve as an effective template for inducing perovskite growth. However, on the Pb-rich and chemically inert amorphous MCP glass surface, lacking the necessary conditions for epitaxial growth, overcoming the nucleation barrier and precisely controlling the crystal facet orientation of TiO_2_ remains a challenge. Current research is mostly limited to phenomenological descriptions, lacking an in-depth physical interpretation of the texture evolution mechanism in a “non-epitaxial growth” environment that combines Surface Energetics.

Based on this, we propose a strategy to construct (101)-oriented TiO_2_ seed layers via surface energetics regulation. We systematically investigated the growth behavior of the TiCl_4_/O_3_ ALD process on MCP glass across a wide temperature range of 150–400 °C. Combining DFT calculations with Wulff construction principles, we confirmed that under high-temperature thermal relaxation, a surface energy minimization mechanism drives the film to grow preferentially along the thermodynamically most stable (101) direction. Through process optimization, a (101)-oriented TiO_2_ seed layer was obtained on the amorphous substrate, which successfully induced the singular orientation growth of BaTiO_3_, effectively suppressing random nucleation on the amorphous substrate.

## 2. Materials and Methods

### 2.1. TiO_2_ Thin Film Deposition

Titanium dioxide (TiO_2_) thin films were deposited on circular lead silicate glass substrates (diameter: 25 mm, thickness: 0.5 mm) specifically designed for MCP applications. Prior to deposition, the substrates were ultrasonically cleaned in ethanol to remove surface contaminants. The deposition process was carried out using a thermal atomic layer deposition (ALD) system manufactured by MNT Micro and Nano Tech Co., Ltd. (Wuxi, China).

Titanium tetrachloride (TiCl_4_) and ozone (O_3_) were employed as the titanium precursor and oxygen source, respectively. High-purity nitrogen (99.999%) served as both the carrier and purge gas. To investigate the temperature-dependent growth behavior, the deposition temperature was varied from 150 °C to 400 °C with an interval of 50 °C.

**Figure 1:**
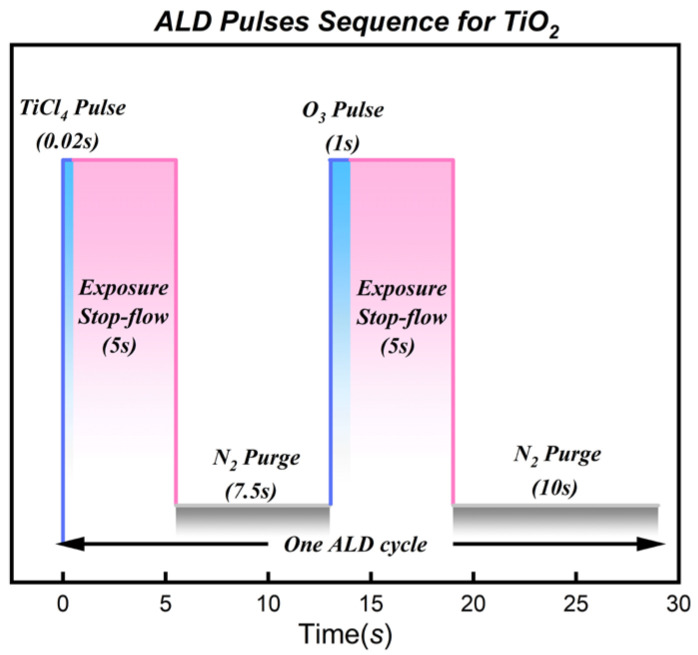
Schematic diagram of the thermal ALD process using TiCl_4_ and O_3_ precursors in exposure mode. The detailed pulse sequence for one full cycle is depicted.

To ensure complete precursor adsorption and saturation of surface reactions within the high aspect ratio structures (simulated here by the exposure process), an “Exposure Mode” was utilized. A single ALD growth cycle consisted of the following sequence: (1) TiCl_4_ pulse (0.02 s); (2) Exposure (5 s); (3) N_2_ purge (7.5 s); (4) O_3_ pulse (1.0 s); (5) Exposure (5 s); (6) N_2_ purge (10 s). The schematic diagram of this ALD reaction cycle is illustrated in Figure 1. The planar glass samples used for the systematic mechanism study were deposited for 1000 cycles. Additionally, to verify process applicability within real high aspect ratio structures, a validation experiment was conducted using a real Microchannel Plate (MCP) substrate (China Building Materials Academy, Beijing, China). The MCP features a pore diameter of 6 μm and an aspect ratio of 40:1. For this specific sample, the deposition temperature was set to the optimized 350 °C, and the number of cycles was increased to 1800 to ensure the film thickness was sufficient for clear cross-sectional morphological observation under SEM.

To ensure the reliability of the results, five parallel glass substrates were loaded into the reaction chamber for each deposition run to verify intra-batch uniformity. The quantitative metrics reported (e.g., GPC, thickness) represent the average values derived from these parallel samples. For spectral characterizations (e.g., XRD patterns, XPS spectra), representative data reflecting the consistent trends observed across the samples are presented. Additionally, independent repetition runs were performed for the critical TiO_2_ seed layer deposition at the optimized temperature (350 °C) to confirm the reproducibility of the preferred orientation.

### 2.2. Template-Assisted Oriented Growth of BaTiO_3_ Layer

To evaluate the efficacy of the optimized TiO_2_ seed layer in modifying the amorphous surface, barium titanate (BaTiO_3_) thin films were subsequently grown in the same ALD reactor. A comparative study was conducted between bare MCP glass substrates and substrates pre-coated with the (101)-oriented TiO_2_ seed layer (fabricated at 350 °C for 1000 cycles).

The BaTiO_3_ films were deposited using a super-cycle strategy involving the alternating deposition of binary oxides. Bis(triisopropylcyclopentadienyl)barium [Ba(iPr_3_Cp)_2_] and TiCl_4_ were employed as the barium and titanium precursors, respectively, with high-concentration ozone (O_3_) serving as the oxidant for both steps. To ensure sufficient vapor pressure for saturative adsorption, the Ba precursor canister was heated to 135 °C.

**Figure 2:**
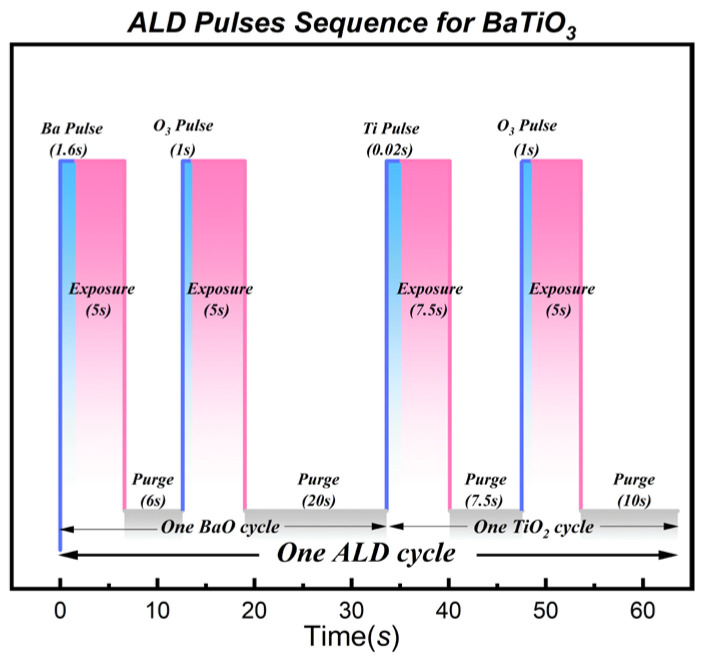
Schematic illustration of the ALD super-cycle sequence for BaTiO_3_ growth. The process involves alternating BaO and TiO_2_ sub-cycles. One full super-cycle consists of a Ba(iPr_3_Cp)_2_/O_3_ sub-cycle followed by a TiCl_4_/O_3_ sub-cycle. The incorporation of exposure steps ensures conformal coverage within the complex MCP structures. The total process repeats for 100 super-cycles to form the oriented film.

To achieve stoichiometric perovskite BaTiO_3_, one ALD “super-cycle” was defined as a sequence of 1 BaO sub-cycle + 1 TiO_2_ sub-cycle. The specific pulse parameters for the BaO sub-cycle were set as follows: (1) Ba source pulse (1.6 s); (2) Exposure (5 s); (3) N_2_ purge (6 s); (4) O_3_ pulse (1.0 s); (5) Exposure (5 s); (6) N_2_ purge (20 s). The parameters for the TiO_2_ sub-cycle remained consistent with those described in Section 2.1. The schematic diagram of this ALD reaction cycle is illustrated in Figure 2. The exposure steps were incorporated to enhance precursor diffusion and adsorption within the high aspect ratio microchannels.

The deposition temperature was maintained at 400 °C, and the total growth process comprised 100 super-cycles. It is noteworthy that due to the limited number of cycles, the resulting films were ultrathin, rendering the XRD signals highly sensitive to the intrinsic crystalline quality. No post-deposition high-temperature annealing was performed to strictly preserve the thermal budget of the MCP glass.

### 2.3. Material Characterization

The crystallographic structure of the thin films was analyzed using a Bruker D8 Advance X-ray diffractometer (XRD, Bruker, Billerica, MA, USA) equipped with a Cu Kα radiation source. A field-emission scanning electron microscope (FE-SEM, Hitachi S-4800, Tokyo, Japan) was employed to examine the surface morphology and cross-sectional microstructure of the films. The surface chemical states were investigated using X-ray photoelectron spectroscopy (XPS, Shimadzu Kratos AXIS Supra, Manchester, UK). All binding energy values were calibrated by referencing the adventitious C 1s peak at 284.8 eV.

### 2.4. Computational Details

Density functional theory (DFT) calculations were performed using the CASTEP module implemented in the Materials Studio 2020 software package [34]. The exchange-correlation energy was treated using the Perdew-Burke-Ernzerhof (PBE) functional within the Generalized Gradient Approximation (GGA) [35]. OTFG ultrasoft pseudopotentials were employed to describe the interactions between electrons and ions, with a plane-wave cutoff energy set to 489.8 eV [36,37]. This cutoff energy aligns with the typical range (380–500 eV) for TiO_2_ calculations as summarized in Table 2 of the recent review by Zavatski et al. [38].

To simulate semi-infinite crystal surfaces, periodic slab models were constructed for the (101), (100), and (001) facets of anatase TiO_2_. A vacuum layer of 15 Å was applied in the direction perpendicular to the surface to eliminate interlayer interactions under periodic boundary conditions. The slab models consisted of three stoichiometric O-Ti-O repeating layers; the bottom layer was fixed to mimic the bulk environment, while the top layers were allowed to fully relax during geometric optimization. According to recent benchmark studies on DFT strategies for TiO_2_ [38], this model thickness is sufficient to screen the interaction between opposite surfaces and capture the correct relative stability trend among different facets. The convergence criteria for geometric optimization were set as follows: energy tolerance < 2.0 × 10^−5^ eV/atom, maximum force < 0.05 eV/Å, and maximum displacement < 0.002 Å.

Specifically, for the preferentially oriented (101) facet, a large supercell containing 108 atoms was constructed to minimize finite-size effects. Given the extensive size of this supercell, Brillouin zone sampling was restricted to the Γ-point (1 × 1 × 1). This sampling strategy is consistent with current research standards for large-scale TiO_2_ systems, as evidenced by the survey of computational parameters in Table 2 of Zavatski et al. [38], where low-density k-point meshes (e.g., 1 × 1 × 1 or 2 × 2 × 1) are commonly adopted for supercell geometries. For the bulk unit cell calculation used to obtain the accurate bulk energy reference ($E_{bulk}$), a 3 × 3 × 3 Monkhorst-Pack grid was employed.

**Figure 3:**
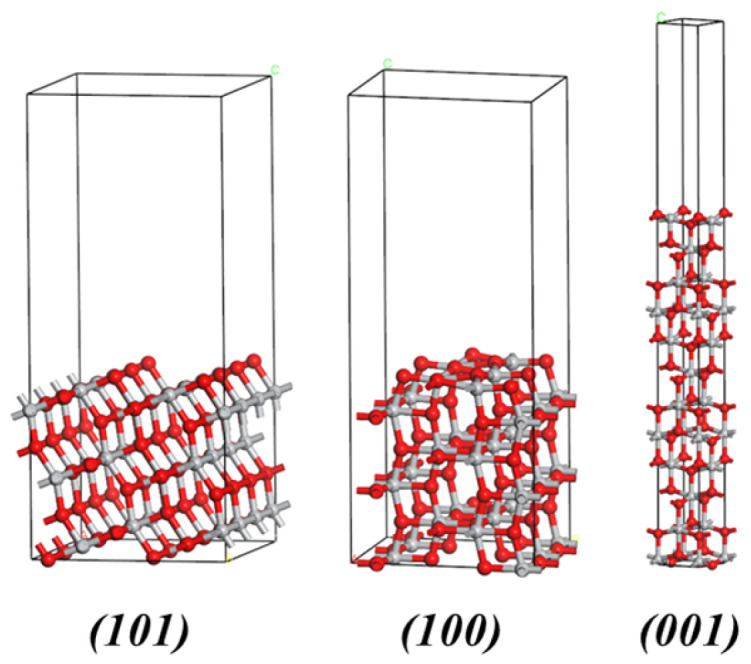
Optimized atomic structures of the anatase TiO_2_ slab models used for surface energy calculations: (left) (101) surface (supercell), (middle) (100) surface, and (right) (001) surface. Gray and red spheres represent Ti and O atoms, respectively.

The surface energy (γ) was calculated using the following equation:

(1) $$\gamma =\frac{E_{slab}-nE_{bulk}}{2A}$$

where $E_{slab}$ is the total energy of the optimized slab model, $E_{bulk}$ is the energy per formula unit of bulk TiO_2_, n is the number of formula units in the slab, and A is the surface area of one side of the slab. The optimized geometries of the slab models are illustrated in Figure 3.

## 3. Results and Discussion

### 3.1. Growth Characteristics and Morphology

**Figure 4:**
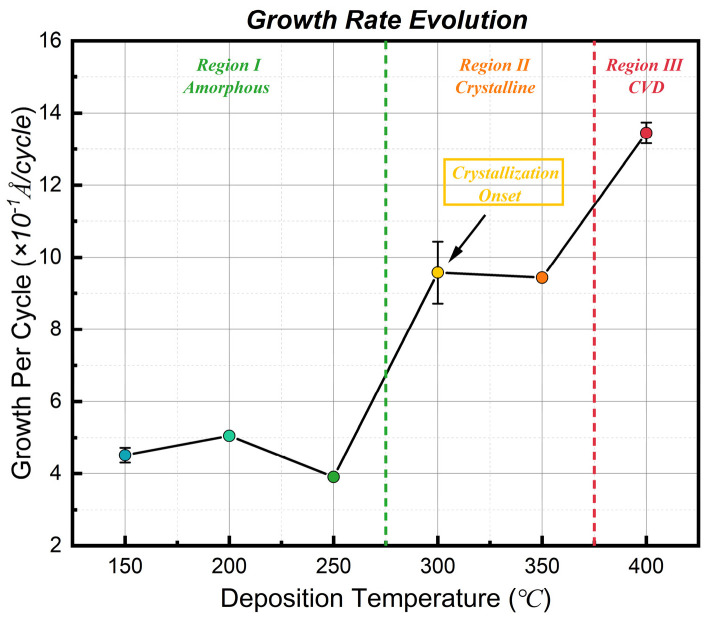
Temperature—dependent growth behavior of ALD TiO_2_ films. The plot shows the Growth Per Cycle (GPC) derived from cross-sectional SEM measurements. (Error bars represent the standard deviation calculated from 4 measurements; in some cases, the error bars are smaller than the data symbols). The growth process is categorized into three regimes: (I) Amorphous region (<300 °C) with kinetically limited growth; (II) Crystalline region (300–350 °C) where GPC increases due to crystallization-induced surface area expansion; and (III) CVD region (400 °C) dominated by parasitic precursor decomposition.

Figure 4 illustrates the variation in the growth per cycle (GPC) of TiO_2_ films as a function of deposition temperature. To identify the optimal process window for MCP seed layers, we categorized the growth behavior into three distinct regimes based on the underlying surface reaction mechanisms.

In Region I (150–250 °C), the GPC exhibits a distinctive “rise-and-fall” evolutionary trend, reaching a minimum of ~0.39 Å/cycle at 250 °C. This non-monotonic behavior reveals a competition between ligand steric hindrance and film densification mechanisms within the low-temperature range [29,30,31,39]. The initial increase in GPC from 150 °C to 200 °C is attributed to the kinetic activation of surface reactions and the elimination of steric hindrance. As evidenced by the XPS data, significant Cl residues exist at 150 °C. These bulky Cl ligands occupy surface active sites, creating strong steric hindrance that blocks the adsorption of subsequent TiCl_4_ precursors, thereby limiting the amount of Ti deposited per cycle. As the temperature rises to 200 °C, enhanced thermal energy promotes ligand exchange reactions, effectively removing surface Cl. The elimination of steric hindrance releases more adsorption sites, increasing the areal density of deposited Ti atoms, which directly leads to the rise in GPC [29,30,31]. However, as the temperature further increases to 250 °C, the GPC decreases. This stage is primarily dominated by the physical densification mechanism. Although reaction efficiency improves at 200 °C, the resulting amorphous film remains in a low-density state (containing physisorbed layers or micropores). At 250 °C, higher thermal energy drives the structural relaxation of the amorphous network and the complete desorption of physisorbed species, causing significant volumetric shrinkage [39]. Therefore, the minimum GPC at 250 °C represents the intrinsic growth rate of the densest, defect-free amorphous TiO_2_. Nevertheless, as shown in the subsequent XRD analysis, films in this regime are amorphous and lack the lattice template function, rendering them unsuitable as effective seed layers for oriented growth.

**Figure 5:**
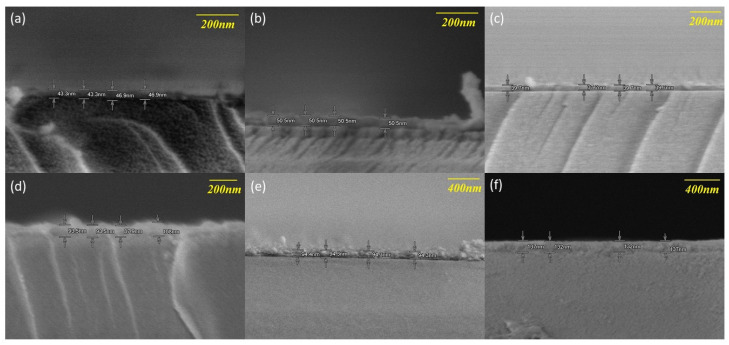
Cross-sectional SEM images of TiO_2_ thin films deposited at various temperatures: (**a**) 150 °C, (**b**) 200 °C, (**c**) 250 °C, (**d**) 300 °C, (**e**) 350 °C, and (**f**) 400 °C. The films deposited at 150–250 °C (**a**–**c**) exhibit a smooth, featureless morphology characteristic of an amorphous structure. The onset of crystallization at 300 °C (**d**) and 350 °C (**e**) leads to a roughened surface with distinct faceted grain structures. At 400 °C (**f**), the film becomes significantly thicker due to parasitic CVD growth, while the surface appears smoother due to enhanced surface diffusion at elevated temperatures.

In Region II (300–350 °C), the GPC jumps to ~0.96 Å/cycle. This sharp increase is closely related to crystallization-induced surface area expansion. The phase transition from amorphous to polycrystalline anatase leads to a surface morphology characterized by faceted grains (Figure 5d,e) [40,41,42,43]. This increase in microscopic roughness is actually a signature of improved crystal quality—indicating the emergence of thermodynamically stable facets (such as the (101) plane). Crucially, although the GPC increases, the process remains primarily controlled by surface chemical reactions (ALD mode). This ensures that precursors can deeply penetrate the microporous structure of MCPs [1,6], making this the ideal window for constructing high-quality seed layers.

In Region III (400 °C), the GPC surges to 1.35 Å/cycle, marking the transition from self-limiting ALD to a parasitic CVD-like regime. This indicates that at such elevated temperatures, thermal energy is sufficient to activate significant surface diffusion of adatoms. The enhanced mobility allows adatoms to migrate laterally, preferentially filling surface depressions, which effectively smoothens the growth front despite the high deposition rate. However, this “smoothness” comes at the cost of the key advantage of ALD: conformality. The non-linear surge in GPC serves as a kinetic indicator that the surface reaction rate has exceeded the limit of surface site saturation. According to reaction-diffusion theory, such rapid surface kinetics in the CVD mode typically lead to precursor depletion near the pore entrances before they can penetrate deep into high aspect ratio structures via Knudsen diffusion [44,45,46]. Therefore, although 400 °C yields higher crystallinity, the kinetic data strongly implies a drastic degradation in step coverage, failing to meet the uniform coating requirements for MCP channels.

**Figure 6:**
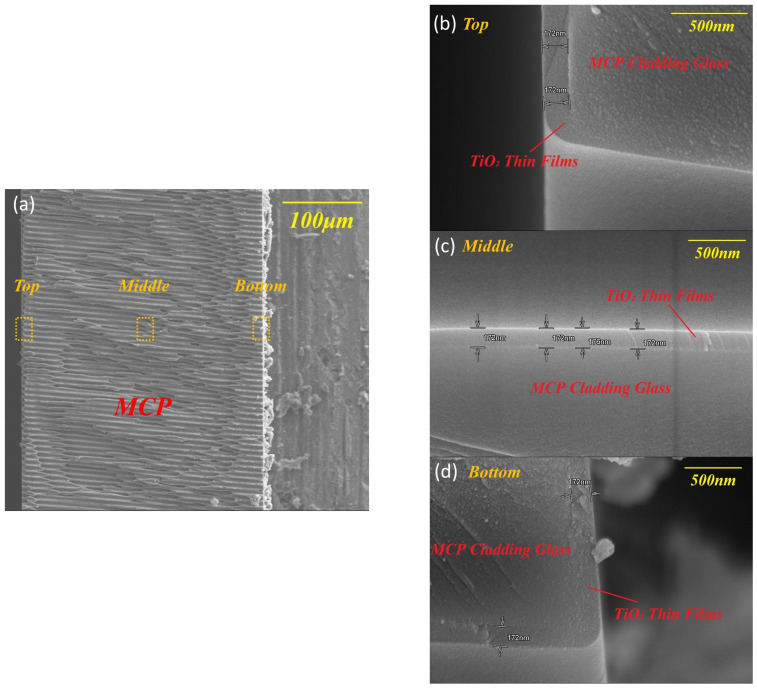
(**a**) Cross-sectional SEM image of a TiO_2_-coated MCP microchannel (deposited at 350 °C for 1800 cycles). High-magnification views of the (**b**) Top, (**c**) Middle, and (**d**) Bottom sections.

To verify the geometric conformality, TiO_2_ was deposited on a real MCP substrate. The deposition was extended to 1800 cycles to facilitate cross-sectional SEM visualization. As shown in Figure 6, the film exhibits exceptional uniformity across the top, middle, and bottom sections of the microchannels, with a consistent thickness of ~172 nm, corresponding to a step coverage of nearly 100%. Notably, the intrapore GPC calculated from this thickness (~0.095 nm/cycle) aligns closely with the planar GPC (~0.094 nm/cycle). This cross-scale consistency strongly attests that the ‘Exposure Mode’ effectively eliminates transport limitations within high aspect ratio structures, achieving ideal self-limiting ALD growth.

### 3.2. Crystallographic Evolution and Texture Analysis

**Figure 7:**
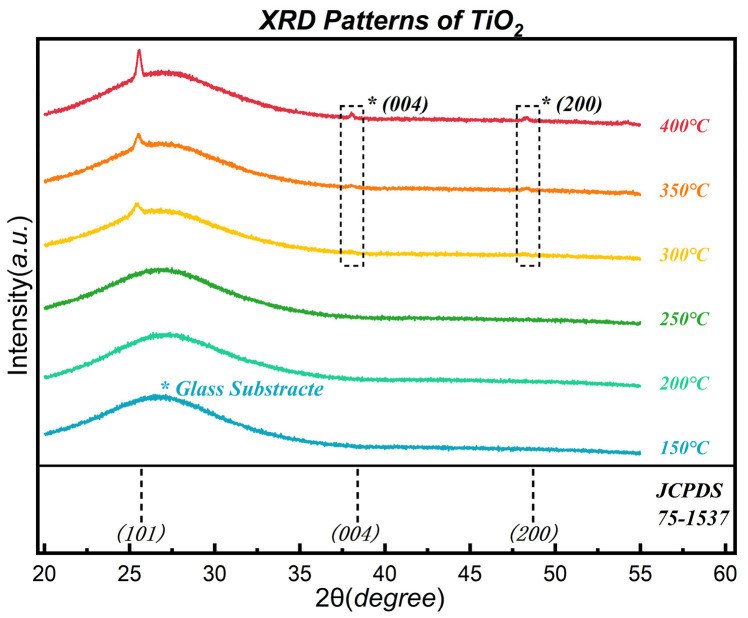
XRD patterns of TiO_2_ thin films deposited at temperatures ranging from 150 °C to 400 °C. The inset displays the evolution of the Full Width at Half Maximum (FWHM) of the (101) peak. Note the exclusive emergence of the (101) reflection at 300 °C, marking the onset of the texture formation required for the seed layer. The asterisks (*) denote the observed scattering features, including the diffraction peaks of anatase TiO_2_ and the broad amorphous hump from the glass substrate.

Figure 7 presents the X-ray diffraction (XRD) patterns of ALD-TiO_2_ thin films deposited across the temperature range of 150–400 °C.

The specific film thicknesses were measured to be 45.1 nm (150 °C), 55.1 nm (200 °C), 39.1 nm (250 °C), 95.7 nm (300 °C), 94.3 nm (350 °C), and 134.5 nm (400 °C). Notably, within the critical window of 300–350 °C, the film thickness reaches ~94 nm, which is sufficient for reliable phase analysis. To rigorously exclude detection limits, we analyzed the signal-to-noise ratio (SNR) based on the 300 °C sample. Referring to the standard card (JCPDS No. 75-1537), the relative intensities of the (200) and (004) reflections for randomly oriented anatase should be 21.4% and 13.8% of the (101) peak, respectively. In our experiment, the (101) peak exhibits a net intensity of ~4000 counts (above the ~3000 counts amorphous background). Theoretically, a random (200) peak would appear with a net intensity of ~850 counts. Given that the statistical noise fluctuation (σ ≈ $\sqrt{N}$) of the background is only ~55 counts, such a secondary reflection would be >15σ above the noise floor. The complete absence of these secondary reflections strongly confirms that the observed singular (101) texture is genuine, ruling out artifacts caused by limited film thickness or XRD noise limits.

In the low-temperature regime (150–250 °C), no characteristic diffraction peaks were detected. The patterns exhibit only a broad hump originating from the amorphous glass substrate. This indicates that within this temperature window, the surface diffusion of precursor adatoms is kinetically limited, resulting in the formation of a disordered amorphous network. Such an amorphous structure lacks the necessary long-range order to serve as an effective lattice template, thereby possessing no capability to induce template-assisted oriented growth of subsequent functional layers.

A critical phase transition occurs when the deposition temperature reaches 300 °C. Notably, a distinct diffraction peak exclusively emerges at 2θ ≈ 25.3°, corresponding to the anatase TiO_2_ (101) plane, while signals from other common facets (such as (004) and (200)) are entirely absent. This “single-peak” characteristic implies that the system spontaneously selects an anisotropic growth path during the nucleation stage.

The physical mechanism underlying this phenomenon can be interpreted on two levels:

First, the absence of the rutile phase aligns with the classical theory by Zhang and Banfield [40], which posits that the anatase phase is thermodynamically stable at the nanoscale.

More critically, regarding the orientation selection within the anatase lattice, this experimental result is highly consistent with our DFT calculations (Section 3.4). Our calculations confirm that among the low-index facets—(101), (100), and (001)—the (101) facet possesses the lowest surface energy. Consequently, during thermal relaxation, the system preferentially drives the nucleation and growth of the (101) plane to minimize the total surface free energy. This thermodynamic screening effect directly leads to the singular (101) texture observed in the experiments.

As the deposition temperature increases further, the crystallization behavior exhibits complex evolution. At 400 °C, although the intensity of the (101) peak increases substantially, weak new diffraction peaks begin to appear near 38.4° (004) and 48.7° (200). The emergence of these secondary facets indicates that excessive thermal energy activates the nucleation of other non-lowest-energy planes, thereby compromising the texture purity of the film to some extent.

To evaluate the crystalline quality of the dominant phase, we focused on the evolution of the Full Width at Half Maximum (FWHM) of the (101) peak (Figure 6). The peak exhibits a significant “sharpening trend” with increasing temperature; the monotonic decrease in FWHM confirms that as the thermal activation energy increases, the defect density within the lattice decreases, and long-range order improves significantly. This suggests that while high temperature introduces trace amounts of stray crystal phases, it effectively promotes the structural refinement of the primary lattice [32,43,47,48,49]. It is worth noting that due to the overlap between the TiO_2_ (101) peak and the broad background signal from the amorphous glass substrate, direct calculation of absolute crystallite size via the Scherrer equation may be subject to artifacts from background subtraction. Therefore, we rely on the relative reduction in FWHM as a robust qualitative indicator of enhanced crystallinity and grain growth, rather than pursuing absolute size quantification. Complementing this qualitative crystallinity analysis, we further quantified the texture evolution using the Lotgering factor (f). The calculated f values are 1.0, 0.863, and 0.680 for films deposited at 300, 350, and 400 °C, respectively. The unity value (f = 1.0) at 300 °C corresponds to the initial pure-phase nucleation. At 350 °C, although the f value decreases slightly to 0.863 due to the emergence of trace secondary facets, it indicates that the (101) orientation remains overwhelmingly dominant. When combined with the sharp FWHM, this confirms that 350 °C represents the optimal trade-off between orientation purity and crystalline quality. Conversely, the significant drop to 0.680 at 400 °C quantitatively reflects the intensified competitive growth and texture degradation at elevated temperatures.

While 300 °C marks the exclusive onset of the (101) texture, its relatively weak diffraction intensity suggests that crystallinity remains in the incipient stage. Conversely, although 400 °C yields the highest crystallinity, it is accompanied by secondary facet impurities and surface degradation caused by the CVD mechanism. The observed texture degradation (i.e., the appearance of secondary peaks) at 400 °C can be attributed to the synergistic interplay of thermal activation and thickness effects. At 400 °C, the film thickness increases to ~133 nm, reducing the surface-to-volume ratio and thereby weakening the absolute dominance of surface energy over crystal growth. Concurrently, the elevated thermal energy enables precursors to overcome the higher nucleation barriers required to form the energetically costlier (004) and (200) facets. Thus, while surface energy minimization dictates the orientation during the initial nucleation stage (300–350 °C), bulk growth kinetics and increased thickness at elevated temperatures tend to induce entropy-driven orientation randomization.

In contrast, the sample deposited at 350 °C demonstrates an optimal balance between crystalline quality and orientation purity. At this temperature, the intensity of the (101) peak is significantly enhanced—implying sufficient lattice order to provide clear diffraction signals—while maintaining extremely high texture purity (free of stray peaks) and a smooth surface morphology. Therefore, 350 °C was selected as the optimal processing parameter for the subsequent oriented growth of BaTiO_3_ (detailed in Section 3.5).

### 3.3. Chemical Composition and Valency State

To gain in-depth insights into the chemical composition, elemental valence states, and surface microstructure evolution of the ALD-TiO_2_ films, X-ray photoelectron spectroscopy (XPS) analysis was performed. All binding energy data were calibrated referencing the C 1s peak at 284.8 eV.

**Figure 8:**
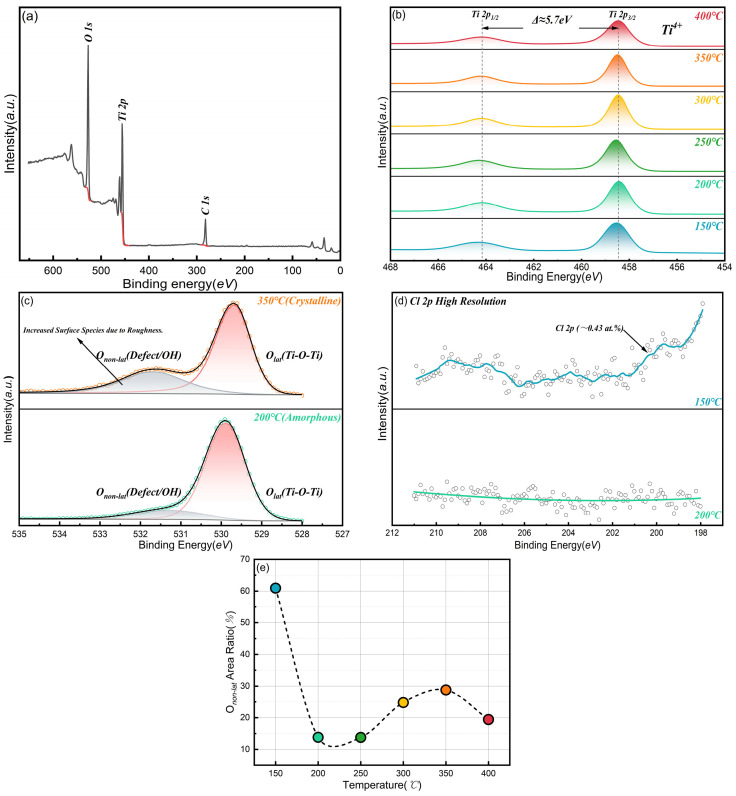
XPS spectra and quantitative analysis of ALD TiO_2_ films. (**a**) Survey scans indicating the presence of Ti, O, Cl, and C. (**b**) High-resolution Ti 2p spectra showing the stable Ti^4+^ state. (**c**) High-resolution O 1s spectra deconvoluted into lattice oxygen (Olat) and non-lattice oxygen (Onon-lat). (**d**) High-resolution Cl 2p spectra, highlighting the complete removal of chlorine impurities at temperatures ≥ 200 °C. (**e**) Quantitative evolution of the non-lattice oxygen ratio (Onon-lat/Ototal) as a function of temperature. The “U-shape” trend is driven by low-density porosity at 150 °C and crystallization-induced roughening at 300–350 °C.

The XPS survey scans (Figure 8a) confirm that the films are composed of Ti and O, accompanied by adventitious carbon contamination. Notably, the evolution of chlorine (Cl) impurities provides critical clues regarding the chemical purity of the films. As detailed in Figure 8d, the film deposited at 150 °C exhibits a distinct Cl 2p signal (~0.43 at.%). This residue is attributed to insufficient thermal energy at low temperatures, which prevents ozone from completely substituting the Cl ligands in the precursor. In sharp contrast, for films deposited at 200 °C and above, the Cl signal drops below the detection limit. This indicates that highly efficient ligand exchange is achieved within the ALD window, ensuring that the seed layer does not introduce lattice distortions or nucleation centers induced by Cl impurities when serving as an epitaxial template [50].

The high-resolution Ti 2p spectra (Figure 8b) display characteristic doublet peaks located at 458.5 eV (2p3/2) and 464.2 eV (2p1/2), with a spin–orbit splitting of 5.7 eV. This confirms that across all deposition temperatures, titanium exists exclusively in the stable Ti^4+^ oxidation state within the TiO_2_ lattice [51,52]. The absence of shoulder peaks associated with Ti^3+^ (oxygen vacancies) suggests that the films possess excellent stoichiometry, which is crucial for maintaining the insulating and dielectric properties of the seed layer.

The O 1s spectra (Figure 8c) reveal the oxygen bonding environment, with the main peak corresponding to the Ti-O-Ti framework and the shoulder peak assigned to surface hydroxyls or adsorbed water. The evolution of the non-lattice oxygen ratio (Onon-lat/Ototal) exhibits a remarkable “U-shaped” trend (Figure 8e), revealing three distinct stages governed by different physical mechanisms:

Steric-hindrance-dominated regime (150 °C): An anomalously high Onon-lat ratio (~60.9%) is observed. Combined with the aforementioned Cl residue, we hypothesize that the bulky residual Cl atoms act as steric hindrance centers, disrupting the formation of a continuous Ti-O network [50]. This results in a “sponge-like” low-density porous structure, making the film highly permeable to atmospheric water molecules. This loose microstructure further proves that low-temperature amorphous films are unsuitable as high-quality seed layers.

Densification transition regime (200–250 °C): With the complete removal of Cl, the ratio drops precipitously to a minimum (~13.8%). This marks the transition of the film into a dense, impurity-free amorphous phase, effectively blocking subsurface moisture absorption.

Crystallization-roughening-dominated regime (300–350 °C): The ratio rebounds, reaching a peak at 350 °C (~28.8%). It must be emphasized that this increase does not stem from a degradation of internal film quality, but rather from a “geometric effect” induced by crystallization. As confirmed by SEM and XRD, the phase transition to polycrystalline anatase induces grain faceting, significantly increasing the specific surface area. These newly exposed (101) facets and inter-granular boundaries become active sites for post-deposition moisture adsorption [53]. Therefore, the high Onon-lat here is driven by crystallization and surface roughening increasing hydroxyl adsorption sites, rather than by lattice defects.

High-temperature CVD and thermal dehydroxylation regime (400 °C): The ratio falls back to ~19.4%. This reduction is highly consistent with the CVD-dominated growth mode and surface planarization observed in Section 3.1. At 400 °C, intense thermal energy triggers two key processes: First is the physical morphology change—enhanced surface diffusion and CVD deposition preferentially fill surface depressions, significantly reducing the specific surface area and thus physically reducing water adsorption sites. Second is chemical thermal dehydroxylation—high temperature promotes surface condensation reactions (2Ti-OH → Ti-O-Ti + H_2_O), resulting in a chemically cleaner surface environment. However, although the oxygen environment here appears “purer,” this does not imply it is the preferred seed layer process, as the CVD mode sacrifices the deep-pore conformality critical for MCPs [44,45,46].

### 3.4. DFT Calculations and Growth Mechanism

To gain deeper insights into the preferred orientation behavior of ALD TiO_2_ films on amorphous lead silicate glass substrates, we employed density functional theory (DFT) calculations to investigate the surface energies of the primary low-index facets of anatase TiO_2_—namely, (101), (100), and (001).

First, geometric optimization was performed on the bulk unit cell of anatase TiO_2_. The calculated lattice constants were a = b = 3.804 Å and c = 9.753 Å. Compared to the standard XRD card (JCPDS No. 75-1537: a = 3.730 Å, c = 9.370 Å), the relative deviations are +2.00% and +4.09%, respectively. This error margin is consistent with the well-known tendency of the GGA-PBE functional to slightly overestimate lattice constants, confirming that the selected exchange-correlation functional and computational parameters are sufficient to accurately describe the ground-state structural properties of TiO_2_, providing a reliable model basis for subsequent surface energy calculations.

**Figure 9:**
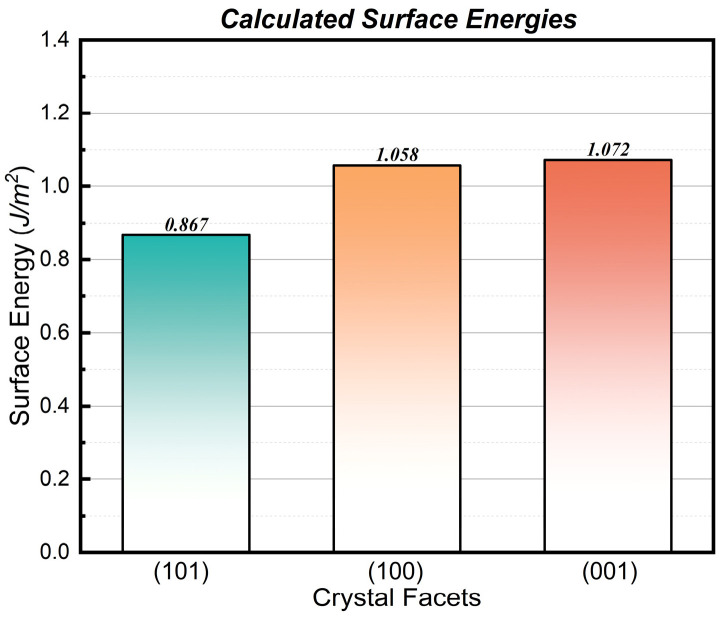
Optimized atomic structures and calculated surface energies of anatase TiO_2_ low-index facets. From left to right: (101), (100), and (001) surfaces. The (101) facet exhibits the lowest surface energy of 0.867 J/m^2^.

Since the glass substrate possesses an amorphous structure, epitaxial growth effects induced by lattice matching are absent. Therefore, under sufficient thermal relaxation, the crystallographic orientation of the film tends to follow the Surface Energy Minimization principle to reach a thermodynamic equilibrium state [40,54]. Figure 9 presents the relaxed surface atomic structural models and the calculated surface energy values. The calculation results indicate that the surface energies (γ) of different facets follow the order:

$$\gamma_{101}\left(0.867\;J/m^{2}\right)<\gamma_{100}\left(1.058\;J/m^{2}\right)<\gamma_{001}\left(1.072\;J/m^{2}\right)$$

Evidently, the (101) facet possesses the lowest surface energy, indicating it is the thermodynamically most stable surface, which aligns with previous theoretical studies [53,55]. According to the Gibbs-Curie-Wulff law, crystal growth always tends to minimize the total surface free energy; thus, facets with lower surface energy will occupy the largest surface area and be preserved during competitive growth [42,56].

Although the initial nucleation process may be influenced by complex kinetic factors (such as nucleation barriers), our DFT results showing that the (101) facet has the lowest surface energy are highly consistent with the classic findings of Yang et al. [42]. They demonstrated that in the absence of surfactant modification, anatase TiO_2_ always tends to expose the thermodynamically most stable (101) facet. Furthermore, according to the thermodynamic model established by Zhang and Banfield [40], for nanomaterials with high surface-area-to-volume ratios (such as the thin films in this study), the contribution of surface energy to the total Gibbs free energy is decisive. Therefore, the experimentally observed trend of enhanced (101) preferred orientation with increasing temperature provides compelling evidence that under high-temperature thermal relaxation conditions, the surface energy minimization mechanism is indeed the plausible criterion governing the texture evolution of the film.

**Figure 10:**
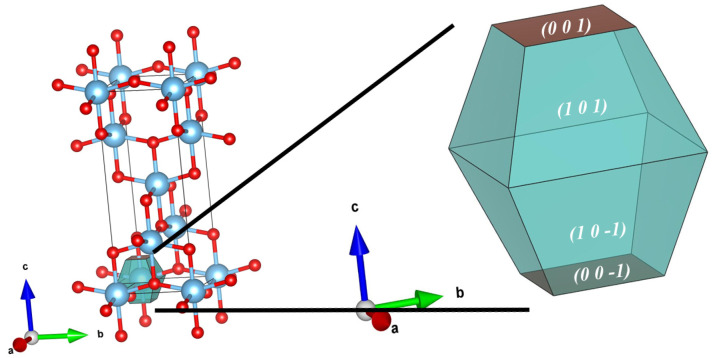
Wulff Equilibrium Crystal Shape (ECS) of anatase TiO_2_. The morphology is constructed based on the calculated surface energies. The thermodynamically stable (101) facets dominate the crystal shape. Light blue and red spheres represent Ti and O atoms, respectively.

To visually demonstrate the impact of surface energy anisotropy on crystal morphology, we constructed the Wulff Equilibrium Crystal Shape (ECS) of anatase TiO_2_ based on the aforementioned calculation results, as shown in Figure 10. The results show that the ideal equilibrium morphology manifests as a typical truncated bipyramid structure.

The (101) facet, with the lowest surface energy, occupies the vast majority of the surface area, constituting the main body of the bipyramid. The (001) facet is located only at the top and bottom, with a smaller area. Notably, although the (100) facet is also a low-index plane, it is geometrically eliminated in the Wulff construction. This indicates that the (100) facet lacks thermodynamic competitiveness relative to the (101) facet and tends to be replaced by more stable facets under equilibrium growth conditions.

It is acknowledged that the DFT models employed here assume ideal, stoichiometric surfaces, whereas the actual ALD process involves complex surface chemistry with adsorbates (e.g., -OH, ligands) and reaction intermediates. Physically, these surface species are expected to passivate undercoordinated surface atoms (dangling bonds), thereby lowering the absolute surface energies. While this stabilization effect might be theoretically more pronounced on high-energy facets (e.g., (001)) due to their higher density of reactive sites—potentially narrowing the stability gaps between different orientations—the intrinsic thermodynamic advantage of the (101) facet remains robust. Consequently, the stability hierarchy ($\gamma_{101}$ < $\gamma_{100}$ < $\gamma_{001}$) is expected to persist even under realistic growth conditions.

This theoretical result, combined with the valid stability hierarchy, correlates highly with our XRD experimental observations (Section 3.2) and elucidates the driving force behind the microstructural evolution:

1. Kinetic Limitation Stage (<300 °C): At low temperatures, the thermal energy of precursor adatoms is insufficient to overcome the surface diffusion barrier. Atoms cannot migrate to thermodynamically equilibrium positions, resulting in an amorphous film state.
2. Thermodynamically Dominated Nucleation Stage (300 °C): When the temperature reaches the crystallization threshold, adatoms acquire sufficient kinetic energy for rearrangement. Since the energy barrier to form the (101) facet is the lowest, the system “exclusively” selects the (101) preferred orientation for growth.
3. Competitive Growth Stage (>300 °C): As the temperature increases further (350–400 °C), higher thermal energy enables atoms to overcome higher energy barriers, allowing the local formation of higher-surface-energy (004) and (200) facets. This thermal activation, combined with the weakened surface energy dominance due to increased film thickness, leads to the emergence of secondary textures and the observed degradation in orientation quality.

### 3.5. Verification of Seed Layer Efficacy: BaTiO_3_ Template-Assisted Oriented Growth

To demonstrate the critical role of the TiO_2_ template in enabling template-assisted oriented growth on amorphous substrates, we conducted systematic comparative deposition experiments. Under identical ALD process conditions at 400 °C, BaTiO_3_ thin films were grown on both bare glass substrates and substrates pre-deposited with the (101)-oriented TiO_2_ seed layer. This section evaluates the specific efficacy of the seed layer through crystallographic evolution and chemical phase analysis.

**Figure 11:**
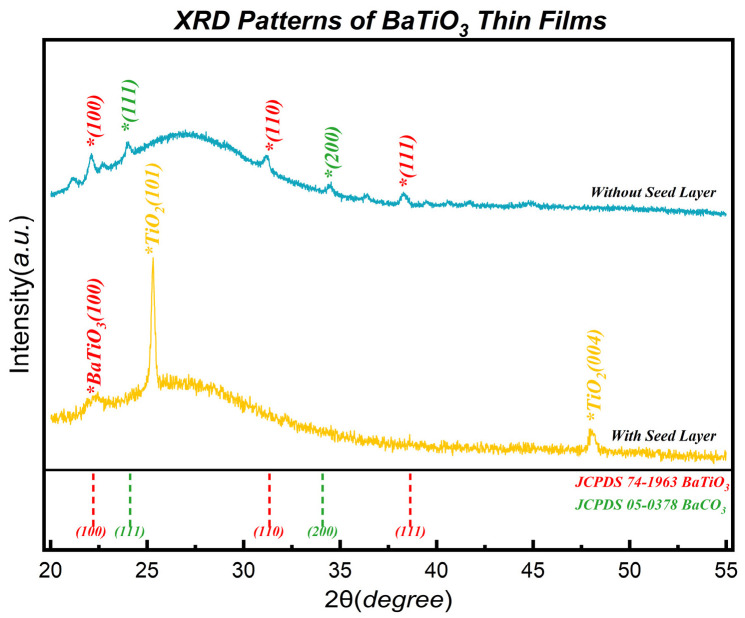
Comparative XRD patterns of BaTiO_3_ thin films grown on bare MCP glass (blue line) and on the TiO_2_-seeded substrate (yellow line). The bare glass substrate leads to random polycrystalline growth with BaCO_3_ impurities, whereas the TiO_2_ seed layer induces singular (100)-oriented template-assisted oriented growth. The asterisks (*) denote the characteristic diffraction peaks of BaCO_3_, BaTiO_3_, and TiO_2_.

Figure 11 presents the XRD patterns, revealing the decisive impact of the substrate surface state on the crystallization behavior.

On the bare glass substrate (blue line), the film exhibits a random nucleation mode. The diffraction pattern displays disordered polycrystalline features, with coexisting (100), (110), and (111) perovskite peaks. More critically, the lack of a structural template leads to significant phase separation, characterized by the appearance of distinct parasitic peaks at 2θ ≈ 23.9° and 33.7°. These peaks correspond to orthorhombic BaCO_3_ (Witherite) impurities. The origin of these impurities lies in the surface chemistry limitations of the substrate. On the amorphous glass surface, the lack of suitable nucleation sites leads to an elongated incubation period and island growth mode, leaving a significant fraction of Ba precursors incompletely reacted or exposed as segregated BaO-like clusters. These active sites readily react with moisture and atmospheric CO_2_ during post-deposition handling [57].

The spectrum is dominated by the underlying anatase TiO_2_ (101) and (004) peaks from the seed layer, superimposed with a singular BaTiO_3_ (100) diffraction peak (2θ ≈ 22.2°). All impurity peaks and randomly oriented diffraction peaks are completely suppressed. The BaTiO_3_ film thickness is estimated to be approximately 20 nm based on the calibrated growth rate. Although the absolute intensity of the diffraction peak is moderate due to this ultrathin nature, the crystallographic data provides robust evidence for singular orientation. According to standard powder diffraction data (JCPDS No. 74-1963), the (110) reflection is the strongest peak (100% intensity) for randomly oriented BaTiO_3_, while the (100) peak is significantly weaker (~20.5%). The clear resolution of the theoretically weaker (100) peak, contrasted with the complete absence of the theoretically strongest (110) peak, rules out the possibility of random orientation masked by detection limits. This specific intensity reversal confirms that the underlying TiO_2_ lattice effectively dominates the nucleation kinetics.

This successful oriented growth can be primarily attributed to the surface chemistry of the seed layer: the (101)-oriented TiO_2_ provides a dense array of reactive Ti-OH/Ti-O termination sites. This promotes the immediate formation of stable Ba-O-Ti bonds, effectively locking the barium ions into the lattice and preventing their reaction with environmental CO_2_. Complementing this chemical stability, the growth is also governed by crystallographic compatibility. Based on the experimental data, the lattice mismatch between anatase TiO_2_ (a ≈ 3.73 Å) and cubic BaTiO_3_ (a ≈ 4.00 Å) is calculated to be approximately 7.2%. This moderate mismatch is accommodated through strain relaxation. Structurally, the characteristic TiO_6_ octahedral chains on the anatase (101) surface provide topological continuity with the perovskite oxygen sublattice, allowing the coordination environment to reorganize from an edge-sharing to a corner-sharing arrangement, thereby physically guiding the (100) orientation. These combined results confirm the critical dual role of the TiO_2_ layer as both a structural template and a chemical passivation layer.

Further insight into the chemical purity of the deposited films was provided by high-resolution C 1s XPS analysis (Figure S1, Supporting Information). While unavoidable adventitious carbonate species (~289 eV) arising from atmospheric exposure were detected on the surfaces of both samples, a striking difference was observed in their relative amounts. The intensity and integrated area of the carbonate signal on the bare glass substrate were drastically higher compared to the TiO_2_-seeded sample. This intense signal is indicative of severe bulk carbonization of unreacted precursors on the bare glass, consistent with the Witherite phase identified in XRD. In strong contrast, the significantly reduced carbonate signal on the seeded sample confirms that the TiO_2_ template effectively promotes the formation of the target perovskite phase while suppressing bulk carbonate impurities.

**Figure 12:**
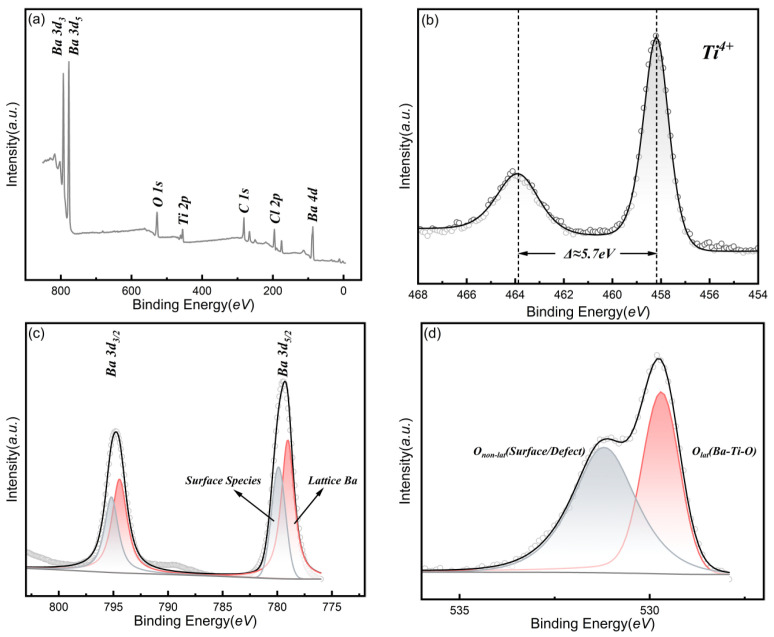
XPS spectra of the BaTiO_3_ thin film grown on the TiO_2_ seed layer. (**a**) Survey scan, (**b**) High-resolution Ti 2p spectrum, (**c**) High-resolution Ba 3d spectrum, and (**d**) High-resolution O 1s spectrum verifying the formation of the metal-oxide lattice.

To further verify the elemental composition and chemical bonding state of the oriented film, detailed XPS analysis was performed on the BaTiO_3_ film grown on the TiO_2_ seed layer (Figure 12).

The survey scan (Figure 12a) confirms the presence of constituent Ba, Ti, and O elements in the film. A trace Cl signal (Cl 2p peak) was detected, which is attributed to residues from incomplete precursor ligand exchange typical of the low-temperature ALD process. However, corroborated by the XRD results where no chlorine-containing impurity phases were identified, it is inferred that these residual Cl atoms do not form long-range ordered independent crystalline phases. Instead, they likely exist as point defects or segregate at grain boundaries, with concentrations below the threshold required to disrupt the host perovskite lattice structure.

Figure 12b shows the high-resolution Ti 2p spectrum. The profile exhibits a highly symmetric doublet structure, with the Ti 2p3/2 and Ti 2p1/2 peaks located at 458.2 eV and 463.9 eV, respectively. The spin–orbit splitting value of 5.7 eV is consistent with the characteristics of the Ti^4+^ oxidation state in standard perovskite BaTiO_3_, ruling out the existence of low-valence defects such as Ti^3+^.

The Ba 3d spectrum (Figure 12c) reveals a complex surface chemical environment. Through Gaussian-Lorentzian peak fitting, the Ba 3d5/2 signal is resolved into two components. The dominant peak at a lower binding energy (~779.0 eV) corresponds to Lattice Ba coordinated by 12 oxygen atoms within the perovskite lattice. The secondary peak (shoulder) at a higher binding energy (~780.0 eV) is attributed to a Surface Restructuring Layer. Due to the high surface activity of barium, surface Ba atoms tend to undergo relaxation or react with residual Cl and atmospheric CO_2_, forming non-perovskite surface termination species. Importantly, the dominance of the lattice barium component confirms that the bulk of the film possesses a well-defined perovskite structure.

Finally, the O 1s spectrum (Figure 12d) provides complementary chemical evidence to substantiate the crystallographic identification of the BaTiO_3_ phase. The spectrum is dominated by a sharp peak at 529.7 eV, assigned to the lattice oxygen (Olat) within the Ba-Ti-O framework. This peak serves as a distinct chemical fingerprint for the formation of the perovskite structure, specifically corresponding to the oxygen anions within the [TiO_6_] octahedral network and Ba-O coordinations. While non-perovskite impurities such as barium carbonate (BaCO_3_)—which appeared on the bare glass substrate—would exhibit oxygen signals at significantly higher binding energies (>531 eV), the clear dominance of the low-binding-energy signal at 529.7 eV confirms that the film is chemically established as a metal-oxide lattice. Consequently, this XPS result corroborates the XRD analysis: the diffraction peak observed at 2θ ≈ 22.1° originates from the crystallized BaTiO_3_ perovskite phase built upon the [TiO_6_] framework, definitely ruling out misidentification with other surface impurity phases.

Consequently, these findings conclusively validate the capability of ALD-grown TiO_2_ films to serve as robust seed layers for BaTiO_3_ template-assisted oriented growth.

## 4. Conclusions

In this work, we systematically investigated the growth behavior of TiO_2_ thin films on glass substrates using TiCl_4_/O_3_ atomic layer deposition (ALD) across a wide temperature window of 150–400 °C. By combining comprehensive experimental characterization with first-principles density functional theory (DFT) calculations, we established a comprehensive understanding ranging from surface chemistry to thermodynamic mechanisms and successfully validated the application of these films in template-assisted oriented growth. The key conclusions are summarized as follows:

1. Chemical Purity and Competitive Evolution of Surface Microstructure:

The study reveals that the O_3_-based process achieves efficient ligand exchange above 200 °C, completely eliminating the ~0.43 at.% chlorine impurities residual at low temperatures (150 °C) and marking the onset of the ideal ALD window. XPS analysis further elucidates that the evolution of non-lattice oxygen (Onon-lat) is governed by two competitive mechanisms: In the low-temperature regime (150 °C), the steric hindrance effect of residual Cl atoms results in a loose porous structure, triggering the physical adsorption of atmospheric moisture (Onon-lat ratio reaching ~61%); whereas in the high-temperature crystallization regime (300–350 °C), the resurgence of Onon-lat is dominated by crystallization-induced surface roughening, where the formation of faceted grains maximizes surface adsorption sites.

2. Thermodynamically Driven Texture Formation Mechanism:

The films exhibit a distinct phase transition from amorphous to polycrystalline anatase at 300 °C, accompanied by an exclusive (101) preferred orientation. DFT calculations provide a robust theoretical basis for this structural evolution, indicating that the (101) facet possesses the lowest surface energy (0.867 J/m^2^). This thermodynamic stability drives the system to follow a surface energy minimization mechanism during thermal relaxation, promoting the preferential growth of (101)-oriented grains, thereby establishing a long-range ordered structure on the lattice-free amorphous substrate.

3. Verification of Seed Layer Efficacy and Optimal Process:

Balancing crystallinity with chemical purity, 350 °C was identified as the optimal processing temperature for fabricating high-quality seed layers. Comparative experiments conclusively demonstrate that the optimized TiO_2_ (101) seed layer effectively overcomes the nucleation barrier on the amorphous surface. In sharp contrast to the random polycrystalline growth and carbonation phenomenon observed on bare glass substrates, the seed layer successfully induced a transition of the subsequent BaTiO_3_ film to singular (100) template-assisted oriented growth. Crucially, the practical applicability of this strategy was validated on real MCP substrates (aspect ratio 40:1). Enabled by the “Exposure Mode,” we achieved near 100% step coverage and uniform thickness distribution within the microchannels. This confirms that the proposed process successfully bridges the gap between precise crystallographic control and stringent geometric conformality requirements, paving the way for the fabrication of high-performance MCP devices.

In summary, this work not only elucidates the complex physicochemical processes governing film growth on amorphous substrates through corroborated experimental and theoretical evidence but also provides a versatile surface engineering pathway for integrating high-performance functional oxides on heat-sensitive devices such as microchannel plates (MCPs).

## Data Availability

The original contributions presented in this study are included in the article. Further inquiries can be directed to the corresponding author.

## Supplementary Materials

The following supporting information can be downloaded at: https://www.mdpi.com/article/10.3390/nano16040281/s1, Figure S1: High-resolution XPS C 1s spectra of BaTiO_3_ thin films deposited on (a) TiO_2_-seeded substrates and (b) bare glass. The peak at ~284.8 eV is attributed to adventitious carbon (C-C/C-H) used for calibration. While both samples show a feature at ~289 eV corresponding to carbonate species, the bare glass sample exhibits a significantly stronger signal, identified as bulk BaCO_3_ (consistent with the Witherite phase in XRD). In contrast, the much weaker signal on the seeded sample is attributed to surface adsorbed carbonates, demonstrating the effective suppression of bulk phase degradation by the TiO_2_ seed layer.

## Abbreviations

The following abbreviations are used in this manuscript:

ALD	Atomic Layer Deposition
MCP	Microchannel Plate
DFT	Density Functional Theory
SEM	Scanning Electron Microscopy
XRD	X-ray Diffraction
XPS	X-ray Photoelectron Spectroscopy
PBE	Perdew–Burke–Ernzerhof
GGA	Generalized Gradient Approximation

## References

[1] Leskovar B. (1977). Microchannel Plates. Phys. Today.

[2] Platzman P.M., Dykman M.I. (1999). Quantum Computing with Electrons Floating on Liquid Helium. Science.

[3] Roth P., Fraser G.W. (2000). Microchannel Plate Resistance at Cryogenic Temperatures. Nucl. Instrum. Methods Phys. Res. A Accel. Spectrom. Detect. Assoc. Equip..

[4] Gorelikov D., Sullivan N., De Rouffignac P., Li H., Narayanamoorthy J., Tremsin A.S. (2014). Development of Atomic Layer Deposition-Activated Microchannel Plates for Single Particle Detection at Cryogenic Temperatures. J. Vac. Sci. Technol. A Vac. Surf. Films.

[5] Cai H., Zhou D., Shi Y., Tang X., Li K., Zhou Y., Zhang Y., Liu H., Shi X., Li Q. (2020). Temperature-Dependent Low Bulk Resistance of Silicate Glass Microchannel Plate for Cryogenic Quantum Computing. Proceedings of the AOPC 2020: Optoelectronics and Nanophotonics; and Quantum Information Technology, Beijing, China, 30 November–2 December 2020.

[6] Mane A.U., Peng Q., Elam J.W., Bennis D.C., Craven C.A., Detarando M.A., Escolas J.R., Frisch H.J., Jokela S.J., McPhate J. (2012). An Atomic Layer Deposition Method to Fabricate Economical and Robust Large Area Microchannel Plates for Photodetectors. Phys. Procedia.

[7] Li X., Cai H., Zhao X., Liu H., Zhu Y., Li K., Huang Y., Ma J., Meng F., Gu J., Zhou L., Guo J. (2025). Gain Regulation of Microchannel Plate via Atomic Layer Deposition of Al_2_O_3_ Films. Proceedings of the AOPC 2025: Optical Devices and Integration, Beijing, China, 24–27 June 2025.

[8] Adams B.W., Elagin A., Elam J.W., Frisch H.J., Genat J.F., Gregar J.S., Mane A.U., Minot M.J., Northrop R., Obaid R. (2015). An Internal ALD-Based High Voltage Divider and Signal Circuit for MCP-Based Photodetectors. Nucl. Instrum. Methods Phys. Res. A Accel. Spectrom. Detect. Assoc. Equip..

[9] Conneely T.M., Milnes J.S., Howorth J. (2013). Extended Lifetime MCP-PMTs: Characterisation and Lifetime Measurements of ALD Coated Microchannel Plates, in a Sealed Photomultiplier Tube. Nucl. Instrum. Methods Phys. Res. A Accel. Spectrom. Detect. Assoc. Equip..

[10] Melikyan Y., Skora T., Komárek T., Noka L., Serebryakov D., Urbáek V. (2019). Load Capacity and Recovery Behaviour of ALD-Coated MCP-PMTs. Nucl. Instrum. Methods Phys. Res. A Accel. Spectrom. Detect. Assoc. Equip..

[11] Tian Y., Lu N., Yang Y., Huang W. (2011). Realization of Neutron Sensitive MCP with ALD Technique. Proceedings of the 2011 IEEE Nuclear Science Symposium Conference Record, Valencia, Spain, 23–29 October 2011.

[12] Thompson C.V. (2000). Structure Evolution During Processing of Polycrystalline Films. Annu. Rev. Mater. Sci..

[13] Fong D.D., Stephenson G.B., Streiffer S.K., Eastman J.A., Auciello O., Fuoss P.H., Thompson C. (2004). Ferroelectricity in Ultrathin Perovskite Films. Science.

[14] Cicconi G., Bosi M., Mezzadri F., Ugolotti A., Cora I., Seravalli L., Tornatzky H., Lähnemann J., Wagner M.R., Bhatt P. (2026). Nucleation and Faceting in (001) r-GeO_2_ Heteroepitaxy on r-TiO_2_ by Metalorganic Vapor Phase Epitaxy. Appl. Surf. Sci..

[15] Wu H., Liu H., Zhou X., Xu G., Wang Y., Lv Y., Feng Z., Long S. (2026). High-Quality β-(Al_x_Ga_1-x_)_2_O_3_ Heteroepitaxy Grown on (010) Ga_2_O_3_ via MOCVD and Transistor Demonstration. Semicond. Sci. Technol..

[16] Wang T., Li Y., Steuer O., Xie C., Shaikh M.S., Heller R., Huang Y., Zhu J., Tian M., Li L. (2026). Heteroepitaxial Growth of β-Ga_2_O_3_ Thin Films on Si (111) Substrates via Interfacial Engineering for Self-Powered Ultraviolet Photodetectors. Appl. Surf. Sci..

[17] Zhou H., Zhang C., Zhang K., Huang Z., Liu F., Zhou M., Gong H., Tang S., Liu W., Wang B. (2025). High Power Density Gallium Nitride Radio Frequency Transistors via Enhanced Nucleation in Heteroepitaxy. Nat. Commun..

[18] Choi H.-Y., Jeon J.D., Kim S.E., Jang S.Y., Sung J.Y., Lee S.W. (2023). Strained BaTiO_3_ Thin Films via In-Situ Crystallization Using Atomic Layer Deposition on SrTiO_3_ Substrate. Mater. Sci. Semicond. Process..

[19] Falmbigl M., Golovina I.S., Plokhikh A.V., Imbrenda D., Podpirka A., Hawley C.J., Xiao G., Gutierrez-Perez A., Karateev I.A., Vasiliev A.L. (2017). BaTiO_3_ Thin Films from Atomic Layer Deposition: A Superlattice Approach. J. Phys. Chem. C.

[20] Liu H., Liu S., Kang J., Yue Y., Jiang X. (2025). Effectively Reduce the Softening Temperature and Enhance the Wettability of Na_2_O-CaO-P_2_O_5_ Glasses for the Sealing of Aluminum Alloys via a Modulation of K_2_O/Na_2_O Ratio. J. Alloys Compd..

[21] Cai H., Sun Y., Zhang X., Zhang L., Liu H., Li Q., Bo T., Zhou D., Wang C., Lian J. (2019). Reduction Temperature-Dependent Nanoscale Morphological Transformation and Electrical Conductivity of Silicate Glass Microchannel Plate. Materials.

[22] Mozhaev P.B., Hansen J.B., Jacobsen C.S. (2026). Structure of High-Tilt YBa_2_Cu_3_O_x_ Thin Films by Pulsed Laser Deposition. Appl. Phys. A.

[23] Silva-Contreras I.J., Gallegos-Sanchez P., De La Cruz W., Contreras-Lopez O.E. (2026). Ultra-Thin p-Type Nickel Oxide Films Grown by Reactive Pulsed Laser Deposition at Room Temperature: Production of a Pn Heterojunction. Appl. Surf. Sci..

[24] An Q., Shi F., Fang H. (2026). Particle Deposition Driven by Plasma Discharge in Magnetron Sputtering of TiO_x_ Compound Targets. Vacuum.

[25] Santos López F.J., Martínez J.R., García Gallegos J.H., De La Torre Medina J., Aranda-Espinoza S., Encinas A., Guerrero A.L. (2026). Study of Magnetic and Structural Properties of Nickel Films Deposited by Magnetron Sputtering Using a Powder Target. Phys. B Condens. Matter.

[26] George S.M. (2010). Atomic Layer Deposition: An Overview. Chem. Rev..

[27] Kim J., Hwang I., Kim B., Lee W., Song J., Jung Y., Yoon C. (2025). Deposition of HfO_2_ by Remote Plasma ALD for High-Aspect-Ratio Trench Capacitors in DRAM. Nanomaterials.

[28] Song J., Shi L., Cui S., Meng L., Zhou Q., Jiang J., Jin C., Hu J., Wen K., Zhou S. (2025). High-Performance GaN-Based Green Flip-Chip Mini-LED with Lattice-Compatible AlN Passivation Layer. Nanomaterials.

[29] Arroval T., Aarik L., Rammula R., Mändar H., Aarik J., Hudec B., Hušeková K., Fröhlich K. (2014). Influence of Growth Temperature on the Structure and Electrical Properties of High-permittivity TiO_2_ Films in TiCl_4_-H_2_O and TiCl_4_-O_3_ Atomic-layer-deposition Processes. Phys. Status Solidi A.

[30] Aarik J., Aidla A., Mändar H., Uustare T. (2001). Atomic Layer Deposition of Titanium Dioxide from TiCl_4_ and H_2_O: Investigation of Growth Mechanism. Appl. Surf. Sci..

[31] Aarik L., Arroval T., Rammula R., Mändar H., Sammelselg V., Aarik J. (2013). Atomic Layer Deposition of TiO_2_ from TiCl_4_ and O_3_. Thin Solid Films.

[32] Jolivet A., Labbé C., Frilay C., Debieu O., Marie P., Horcholle B., Lemarié F., Portier X., Grygiel C., Duprey S. (2023). Structural, Optical, and Electrical Properties of TiO_2_ Thin Films Deposited by ALD: Impact of the Substrate, the Deposited Thickness and the Deposition Temperature. Appl. Surf. Sci..

[33] Lazzeri M., Vittadini A., Selloni A. (2001). Structure and Energetics of Stoichiometric TiO_2_ Anatase Surfaces. Phys. Rev. B.

[34] Clark S.J., Segall M.D., Pickard C.J., Hasnip P.J., Probert M.I.J., Refson K., Payne M.C. (2005). First Principles Methods Using CASTEP. Z. Krist.-Cryst. Mater..

[35] Perdew J.P., Burke K., Ernzerhof M. (1996). Generalized Gradient Approximation Made Simple. Phys. Rev. Lett..

[36] Pfrommer B.G., Côté M., Louie S.G., Cohen M.L. (1997). Relaxation of Crystals with the Quasi-Newton Method. J. Comput. Phys..

[37] Vanderbilt D. (1990). Soft Self-Consistent Pseudopotentials in a Generalized Eigenvalue Formalism. Phys. Rev. B.

[38] Zavatski S., Neilande E., Bandarenka H., Popov A., Piskunov S., Bocharov D. (2024). Density Functional Theory for Doped TiO_2_: Current Research Strategies and Advancements. Nanotechnology.

[39] Matero R., Rahtu A., Ritala M., Leskel M., Sajavaara T. (2000). Effect of Water Dose on the Atomic Layer Deposition Rate of Oxide Thin Films. Thin Solid Films.

[40] Zhang H., Banfield J.F. (1998). Thermodynamic Analysis of Phase Stability of Nanocrystalline Titania. J. Mater. Chem..

[41] Iyasu T., Tamura K., Shimizu R., Vlaicu M.A., Yoshikawa H. (2006). Experimental and Theoretical Studies on X-Ray Induced Secondary Electron Yields in Ti and TiO_2_. Appl. Surf. Sci..

[42] Yang H.G., Sun C.H., Qiao S.Z., Zou J., Liu G., Smith S.C., Cheng H.M., Lu G.Q. (2008). Anatase TiO_2_ Single Crystals with a Large Percentage of Reactive Facets. Nature.

[43] Cheng H.-E., Hsu C.-M., Chen Y.-C. (2009). Substrate Materials and Deposition Temperature Dependent Growth Characteristics and Photocatalytic Properties of ALD TiO_2_ Films. J. Electrochem. Soc..

[44] Kondo T., Sawada Y., Akiyama K., Funakubo H., Kiguchi T., Seki S., Wang M.H., Uchida T. (2008). Step Coverage Study of Indium-Tin-Oxide Thin Films by Spray CVD on Non-Flat Substrates at Different Temperatures. Thin Solid Films.

[45] Momose T., Sugiyama M., Kondoh E., Shimogaki Y. (2010). Step Coverage Quality of Cu Films by Supercritical Fluid Deposition Compared with Chemical Vapor Deposition. Jpn. J. Appl. Phys..

[46] Choolakkal A.H., Niiranen P., Dorri S., Birch J., Pedersen H. (2024). Competitive Co-Diffusion as a Route to Enhanced Step Coverage in Chemical Vapor Deposition. Nat. Commun..

[47] Aghaee M., Maydannik P.S., Johansson P., Kuusipalo J., Creatore M., Cameron D.C. (2015). Low Temperature Temporal and Spatial Atomic Layer Deposition of TiO_2_ Films. J. Vac. Sci. Technol. A.

[48] Huang Y., Pandraud G., Sarro P.M. (2013). Characterization of Low Temperature Deposited Atomic Layer Deposition TiO_2_ for MEMS Applications. J. Vac. Sci. Technol. A.

[49] Koca K., Benkli Y.E. (2025). Microstructural and Elemental Characterization of Pumice–Bauxite–Clay Ceramics: Effects of Sintering Temperature and Scherrer-Based Analysis. J. Aust. Ceram. Soc..

[50] Aarik J., Aidla A., Uustare T., Sammelselg V. (1995). Morphology and Structure of TiO2 Thin Films Grown by Atomic Layer Deposition. J. Cryst. Growth.

[51] Biesinger M.C., Lau L.W.M., Gerson A.R., Smart R.S.C. (2010). Resolving Surface Chemical States in XPS Analysis of First Row Transition Metals, Oxides and Hydroxides: Sc, Ti, V, Cu and Zn. Appl. Surf. Sci..

[52] McCafferty W. (1998). Determination of the Concentration of Surface Hydroxyl Groups on Metal Oxide Films by a Quantitative XPS Method. Surf. Interface Anal..

[53] Diebold U. (2003). The Surface Science of Titanium Dioxide. Surf. Sci. Rep..

[54] Hanaor D.A.H., Sorrell C.C. (2011). Review of the Anatase to Rutile Phase Transformation. J. Mater. Sci..

[55] Ying Y., Qing F., Weihua W., Yin W. (2013). First-Principle Study on the Electronic and Optical Properties of the Anatase TiO_2_ (101) Surface. J. Semicond..

[56] Herring C. (1951). Some Theorems on the Free Energies of Crystal Surfaces. Phys. Rev..

[57] Hudson L.T., Kurtz R.L., Robey S.W., Temple D., Stockbauer R.L. (1993). Photoelectron Spectroscopic Study of the Valence and Core-Level Electronic Structure of BaTiO_3_. Phys. Rev. B.

