# Supplementary material for: Constructing (101)-Oriented Anatase TiO2 Seed Layers on Amorphous Microchannel Plate Glass: Surface Energetics and Template-Assisted Oriented Growth
Source: Nanomaterials (Basel). 2026 Feb 23;16(4):281. doi: 10.3390/nano16040281 (PMC12942982; doi:10.3390/nano16040281)
Supplement: Supplementary file 1 [file nanomaterials-16-00281-s001.zip › nanomaterials-4145905-supplementary.pdf]

Supplementary Materials:

Constructing (101)-Oriented Anatase TiO2 Seed Layers on Amorphous Microchannel Plate Glass: Surface Energetics and Template-Assisted Oriented Growth

Xiang Li 1,2,3, Hua Cai 2,3, *, Wei Wang 2,3, Xuan Zhao 2,3, Xin-Yue Guo 2,3, Meng-Nan Ma 2,3, Yue-Yang Zhu 1 and Kai-Ming Li 1 and Hui Liu 2,3

1 School of Physical Science and Technology, Guangxi University, Nanning 530004, China; lx2019212911@163.com (X.L.)

2 China Building Materials Academy, Beijing 100024, China

3 Key Laboratory of China Building Materials Industry for Special Photoelectric Materials,

Beijing 100024, China

*** Correspondence: shuangyaqing@163.com**

$BaTiO_3$ XPS Analysis of Surface Chemical States：To further investigate the chemical nature of the impurity phase observed in XRD and to verify the suppression effect of the seed layer, high-resolution X-ray Photoelectron Spectroscopy (XPS) was performed. **Figure S1** compares the C 1s core-level spectra of $BaTiO_3$ films deposited on bare glass and TiO_2-seeded substrates. As shown in **Figure S1**, the C 1s spectra for both samples exhibit a characteristic peak at approximately 289.0 eV, which is attributed to carbonate species (–CO_3). The presence of this signal on the TiO_2-seeded sample is expected, originating from unavoidable surface adsorption of atmospheric CO_2 during ex-situ sample handling and transfer. However, a **striking contrast in peak intensity** is observed between the two samples. The carbonate peak on the bare glass substrate is significantly more intense and broader compared to that on the seeded sample. This strong signal correlates well with the crystalline Witherite ($BaCO_3$) phase identified by XRD, indicating that the reaction on the bare glass surface extends beyond the topmost atomic layers, resulting in severe **bulk carbonization**. Conversely, the substantially suppressed intensity on the TiO_2-seeded sample suggests that the carbonate species are confined primarily to the surface contamination layer. This confirms that the TiO_2 seed layer effectively promotes the formation of stable Ba-O-Ti bonds, kinetically outcompeting the bulk carbonization pathway.

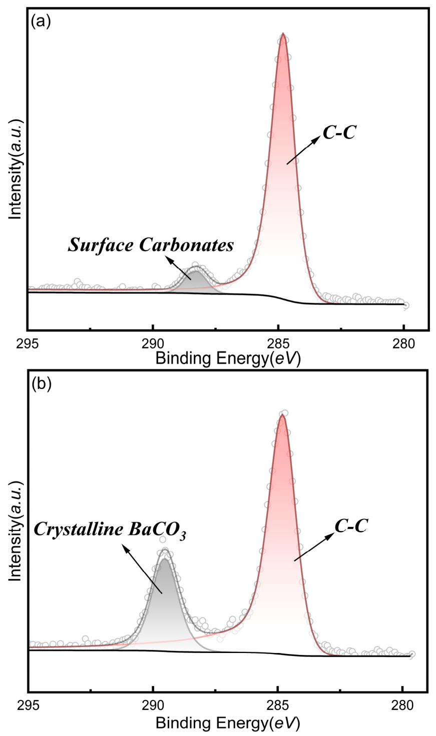

Figure S1. High-resolution XPS C 1s spectra of $BaTiO_3$ thin films deposited on (a) TiO_2-seeded substrates and (b) bare glass. The peak at ~284.8 eV is attributed to adventitious carbon (C-C/C-H) used for calibration. While both samples show a feature at ~289 eV corresponding to carbonate species, the bare glass sample exhibits a significantly stronger signal, identified as bulk $BaCO_3$ (consistent with the Witherite phase in XRD). In contrast, the much weaker signal on the seeded sample is attributed to surface adsorbed carbonates, demonstrating the effective suppression of bulk phase degradation by the TiO_2 seed layer.
